# Digital Monitoring and Management of Patients With Advanced or Metastatic Non-Small Cell Lung Cancer Treated With Cancer Immunotherapy and Its Impact on Quality of Clinical Care: Interview and Survey Study Among Health Care Professionals and Patients

**DOI:** 10.2196/18655

**Published:** 2020-12-21

**Authors:** Oliver Schmalz, Christine Jacob, Johannes Ammann, Blasius Liss, Sanna Iivanainen, Manuel Kammermann, Jussi Koivunen, Alexander Klein, Razvan Andrei Popescu

**Affiliations:** 1 Department of Haematology Oncology and Palliative Care Helios Universitätsklinikum Wuppertal Wuppertal Germany; 2 The Faculty of Business and Law Anglia Ruskin University Cambridge United Kingdom; 3 School of Business University of Applied Sciences Northwestern Switzerland Brugg Switzerland; 4 Medical Affairs (Personalised Healthcare and Patient Access) F Hoffmann-La Roche Ltd Basel Switzerland; 5 Department of Oncology and Radiotherapy Medical Research Center Oulu Oulu University Hospital Oulu Finland; 6 Arcondis AG Reinach Switzerland; 7 Tumor Zentrum Aarau Hirslanden Klinik Aarau Aarau Switzerland

**Keywords:** advanced or metastatic non-small cell lung cancer, cancer immunotherapy, digital patient monitoring, drug- and indication-specific cancer immunotherapy module, eHealth, mHealth, quality of patient care, patient-reported outcomes, real-time symptom reporting, user experience

## Abstract

**Background:**

Cancer immunotherapy (CIT), as a monotherapy or in combination with chemotherapy, has been shown to extend overall survival in patients with locally advanced or metastatic non-small cell lung cancer (NSCLC). However, patients experience treatment-related symptoms that they are required to recall between hospital visits. Digital patient monitoring and management (DPMM) tools may improve clinical practice by allowing real-time symptom reporting.

**Objective:**

This proof-of-concept pilot study assessed patient and health care professional (HCP) adoption of our DPMM tool, which was designed specifically for patients with advanced or metastatic NSCLC treated with CIT, and the tool’s impact on clinical care.

**Methods:**

Four advisory boards were assembled in order to co-develop a drug- and indication-specific CIT (CIT+) module, based on a generic CIT DPMM tool from Kaiku Health, Helsinki, Finland. A total of 45 patients treated with second-line single-agent CIT (ie, atezolizumab or otherwise) for advanced or metastatic NSCLC, as well as HCPs, whose exact number was decided by the clinics, were recruited from 10 clinics in Germany, Finland, and Switzerland between February and May 2019. All clinics were provided with the Kaiku Health generic CIT DPMM tool, including our CIT+ module. Data on user experience, overall satisfaction, and impact of the tool on clinical practice were collected using anonymized surveys—answers ranged from 1 (low agreement) to 5 (high agreement)—and HCP interviews; surveys and interviews consisted of closed-ended Likert scales and open-ended questions, respectively. The first survey was conducted after 2 months of DPMM use, and a second survey and HCP interviews were conducted at study end (ie, after ≥3 months of DPMM use); only a subgroup of HCPs from each clinic responded to the surveys and interviews. Survey data were analyzed quantitatively; interviews were recorded, transcribed verbatim, and translated into English, where applicable, for coding and qualitative thematic analysis.

**Results:**

Among interim survey respondents (N=51: 13 [25%] nurses, 11 [22%] physicians, and 27 [53%] patients), mean rankings of the tool’s seven usability attributes ranged from 3.2 to 4.4 (nurses), 3.7 to 4.5 (physicians), and 3.7 to 4.2 (patients). At the end-of-study survey (N=48: 19 [40%] nurses, 8 [17%] physicians, and 21 [44%] patients), most respondents agreed that the tool facilitated more efficient and focused discussions between patients and HCPs (nurses and patients: mean rating 4.2, SD 0.8; physicians: mean rating 4.4, SD 0.8) and allowed HCPs to tailor discussions with patients (mean rating 4.35, SD 0.65). The standalone tool was well integrated into HCP daily clinical workflow (mean rating 3.80, SD 0.75), enabled workflow optimization between physicians and nurses (mean rating 3.75, SD 0.80), and saved time by decreasing phone consultations (mean rating 3.75, SD 1.00) and patient visits (mean rating 3.45, SD 1.20). Workload was the most common challenge of tool use among respondents (12/19, 63%).

**Conclusions:**

Our results demonstrate high user satisfaction and acceptance of DPMM tools by HCPs and patients, and highlight the improvements to clinical care in patients with advanced or metastatic NSCLC treated with CIT monotherapy. However, further integration of the tool into the clinical information technology data flow is required. Future studies or registries using our DPMM tool may provide insights into significant effects on patient quality of life or health-economic benefits.

## Introduction

Lung cancer was the most common, newly diagnosed malignancy and leading cause of cancer-related death worldwide in 2018 [1]; approximately 85% of cases are classified as non-small cell lung cancer (NSCLC) [2]. Guidelines for patients with locally advanced or metastatic NSCLC without alterations in *EGFR* (epidermal growth factor receptor), *ALK* (anaplastic lymphoma kinase), *ROS1* (ROS proto-oncogene 1), *BRAF* (B-Raf proto-oncogene), *NTRK* (neurotrophic tropomyosin receptor kinase), *RET* (rearranged during transfection), or *MET* (N-methyl-N'-nitroso-guanidine human osteosarcoma transforming) genes, for which targeted therapies are available, recommend first-line treatment with the cancer immunotherapy (CIT) pembrolizumab, as monotherapy for patients with 50% or higher tumor cell programmed death-ligand 1 (PD-L1)–positivity or in combination with chemotherapy [3,4]. Atezolizumab plus bevacizumab, paclitaxel, and carboplatin is also indicated for first-line treatment of patients with metastatic NSCLC and any PD-L1 expression level [5]. For patients who have not previously received CIT treatment, second-line nivolumab, pembrolizumab, or atezolizumab may be given following first-line chemotherapy [3,4].

CIT regimens activate the immune system against cancer and have been shown to slow disease progression and extend overall survival (OS) versus standard chemotherapy and when added to standard chemotherapy [6-13]. However, many patients treated with CIT experience related side effects, such as fatigue, skin rash or itching, diarrhea, nausea, vomiting, dyspnea, and cough [14], in addition to NSCLC-related symptoms [15]. These symptoms can be identified and reported by patients during clinic visits as per the National Cancer Institute (NCI) Common Terminology Criteria for Adverse Events (CTCAE). This patient-reported outcome (PRO) collection has been shown to improve patient-clinician communication, improve patient satisfaction, and enable early symptom detection [16-18]. This has led to the development of PRO-CTCAE, a measurement system designed by the NCI as a companion to the CTCAE to evaluate symptom toxicity [19]. However, the need to rely on patients to recall symptom type and severity over a certain time period between visits can lead to health care professionals (HCPs) receiving incomplete information, thus preventing efficient management.

Digital patient monitoring and management (DPMM) tools may improve clinical practice by allowing patients to report symptoms in real time, enabling direct patient-HCP communication and providing access to patient support materials [20]. However, in addition to collecting and aggregating symptom information weekly, they can also improve patient OS and quality of life (QoL), as well as offer health-economic benefits, such as reduced hospital admission rates and unscheduled visits [21-23]. A study of patients with advanced nonprogressive stage IIA-IV lung cancer finishing first-line chemotherapy found a significantly improved median OS with web-based symptom monitoring versus standard scheduled imaging after a 2-year follow-up: 22.5 versus 14.9 months (hazard ratio 0.59, 95% CI 0.37-0.96, *P*=.03) [24]. To achieve these benefits, DPMM tools must be adopted easily into clinical practice and used frequently so that critical symptoms are reported and detected, and care initiated as early as possible, which is particularly important for increasing OS [25].

In this proof-of-concept pilot study, we assessed factors influencing patient and HCP adoption of our DPMM tool, designed and developed specifically for patients with CIT-treated advanced or metastatic NSCLC, and the impact of such adoption on the quality of clinical care. The study focused on patients treated with CIT due to the high unmet medical need for early detection of critical symptoms in this subgroup. Our tool was based on the generic CIT DPMM tool developed by Kaiku Health in Helsinki, Finland, including all basic features, as well as additional drug-specific features. The Kaiku Health platform was selected due to Kaiku Health’s focus on oncology, including CIT; its market availability in five European Union countries and Switzerland; and its established use in routine practice.

## Methods

### Recruitment

HCP and patient participants were recruited from 10 clinics across Germany, Switzerland, and Finland between February and May 2019. This study used purposive sampling; potential participants with tool experience were selected so that they could provide in-depth information about the research topic [26,27]. Roche developed paper-based materials to support oncologists with patient recruitment, including a welcome letter and device-specific instructions for the platform. Of the 10 clinics involved in the pilot study, three were already using Kaiku Health’s generic CIT DPMM tool and seven had only limited experience with other DPMM platforms. Participating clinics ranged from small community clinics to large university hospitals to reflect the natural diversity of cancer care centers. A single point of contact within each clinic decided on the exact number of HCP participants. A total of 56 patients with advanced or metastatic NSCLC treated with second-line single-agent CIT (ie, atezolizumab or otherwise) were recruited. The final number of included patients was 45, 9 (20%) of whom were treated with atezolizumab; out of 56 patients originally recruited, 5 (9%) declined the invitation and 6 (11%) withdrew early due to disease progression.

### Developing a Drug- and Indication-Specific CIT Module

A literature review was conducted to define CIT-related symptoms and to identify key factors influencing DPMM tool use. Four separate advisory boards were also assembled, with meetings conducted in November 2018 to explore expectations, perceived value, and concerns of HCPs and patients with regard to DPMM tools; these included two boards for physicians (oncologists; n=4), one for nurses (n=4), and one for patients (n=1). The information obtained was used to co-develop a drug- and indication-specific CIT (CIT+) module for Kaiku Health’s DPMM platform, centering on patients’ and HCPs’ needs (see Multimedia Appendix 1). Kaiku Health, as the technical partner, provided the existing, generic CIT DPMM tool, which was used to co-develop the CIT+ module. Kaiku Health’s generic CIT DPMM tool, including the CIT+ module, was provided to the 10 participating clinics, being made available on smartphones, tablets, and desktop computers. Patients treated with a CIT other than atezolizumab had access to functions such as a symptom questionnaire, as per PRO-CTCAE, with 18 questions specific for NSCLC CIT monotherapy; direct message communication between patients and HCPs; indication-specific educational material with information on mild to moderate symptoms and their management [28,29]; and a symptom overview and alerts for HCPs. Patients treated with atezolizumab had access to the above functions and additional drug-specific educational material (eg, a patient card and information on preparing for first infusion and treatment and the likelihood of symptom incidence). Symptom reporting within the tool was required weekly as per the NCI PRO-CTCAE guidelines and based on recommendations from HCPs and patients and usage frequency in seminal clinical trials [22,30]. On-site onboarding sessions of 2 hours in length were held at each clinic between February and April 2019 to train care teams on the CIT+ module and to address questions (see Multimedia Appendix 2). Training included overviews of the pilot study, the partner Kaiku Health, the platform, and triaging workflow. Practical role-based training with simulations, including patient onboarding, electronic PRO (ePRO) form-filling, triaging results, and care team–patient communication, were provided.

### Data Collection

To test the DPMM tool in a real-world setting, data on user experience, overall satisfaction, and the impact of the tool on clinical practice were collected using anonymized surveys and HCP interviews. Fidelity of delivery of the tool and patient adherence were measured using self-reported data in the survey and system usage statistics obtained from the tool itself. Patient surveys were provided to patients by HCPs. An online interim survey consisting of a short questionnaire with 11 closed-ended multiple-choice or Likert scale questions in English, Finnish, or German was conducted after 2 months of tool use to assess user satisfaction and to allow early identification of potential issues. At study end (ie, ≥3 months of tool use), a second online survey consisting of a long questionnaire with 34 and 36 closed-ended multiple-choice or Likert scale questions for patients and HCPs, respectively, was conducted to assess value and to highlight potential gaps or need for improvement. Surveys were built using SurveyMonkey. Semistructured interviews with HCPs in English, Finnish, or German were also conducted at study end to answer 14 open-ended questions to better understand their views on the tool and to increase understanding of survey results. Only a subset of HCPs and patients in each clinic responded to the surveys and interviews. The questions in each were informed by factors included in the original technology acceptance model (TAM) [31], notably perceived usefulness and perceived ease of use [32]. The TAM was selected for its simplicity and for being one of the most commonly used frameworks for assessing user acceptance of new technologies in general health care [33-36] and, specifically, mobile health [37]. The questions were also informed by other elements frequently reported in the literature as influencing adoption of mobile health solutions, for example, patient-clinician communications [38-41], quality of care [42-46], empowerment of patients and care teams [47-53], and efficiency [54-57]. The surveys and interview guides were tested and piloted before their use in the study.

### Data Analysis

Due to the small number of included patients with advanced or metastatic NSCLC treated with atezolizumab (9/45, 20%), data for the whole CIT+ module, including both the generic CIT and the atezolizumab-specific components, were pooled. Survey data were aggregated and analyzed quantitatively using Microsoft Excel for Mac 2011, version 14.4.3 (Microsoft Corporation), to calculate totals, percentages, means, and standard deviations. HCP interviews were recorded, transcribed verbatim, and translated into English, where applicable, for coding. NVivo (QSR International) version 12.6.0 (3841), a qualitative data analysis software package, was used for coding and categorization of transcripts. Data were systematically analyzed using thematic analysis methodology; after the initial analysis and coding by CJ, this was reviewed by MK, and any cases of disagreement were discussed in conjunction with AK and mutually agreed upon (see Multimedia Appendix 3) [58,59]. Anonymized data regarding in-module activities of all included patients, including time to complete the symptoms questionnaire, use of the chat function, and engagement with educational material, were collected by Kaiku Health and shared with Roche through Chartio, software that allows for multiple individuals to access and modify data from different sources. Chartio was used to create detailed usage reports on deidentified user interactions, including log-in events, article reading times, and downloads. The report data were visualized using Data Studio from G Suite Business Solutions (Google).

### Ethical Considerations

Due to the user experience nature of the study, ethics committee approval was not required for the participating sites; however, some sites submitted the study to the ethical committee on a voluntary basis and received approval. Data were anonymized, and no internet protocol data were collected. All participants gave written informed consent. Patients were contacted by their own care team only, and all treatment-related decisions were made solely by the treating physician.

## Results

### User Acceptance of the DPMM Tool and Overall Satisfaction

All user groups, particularly HCPs, showed an increased preference for the desktop version of the tool (see Multimedia Appendix 4). A total of 51 respondents—13 (25%) nurses, 11 (22%) physicians, and 27 (53%) patients—completed the interim survey. Respondents were asked to rank the usability attributes of the tool, with answers rated from 1 (low agreement) to 5 (high agreement). All attributes were ranked quite highly, with mean rank scores ranging from 3.2 to 4.4 for nurses, 3.7 to 4.5 for physicians, and 3.7 to 4.2 for patients (see Table 1). Across all user groups, the highest-ranking attributes were usefulness and communication, followed by ease of use, the value of onboarding, improved quality of care, empowerment, and efficiency (see Table 1). Efficiency was ranked lowest by nurses and physicians, and second lowest by patients; however, overall user acceptance was high and there was a high level of satisfaction with the tool across all user groups. For most user groups, both experienced and new, SDs were less than 1, indicating general alignment. Slight disagreement occurred for new physician users regarding usefulness of the tool (SD 1.2), communication (SD 1.0), and quality of care (SD 1.0).

**Table 1:** Digital patient monitoring and management tool user satisfaction among interim survey respondents.

Usability attribute	Rating^a^, mean (SD)		
	Nurses (n=13)	Physicians (n=11)	Patients (n=27)
Onboarding	4.0 (0.5)	4.1 (1.0)	4.1 (0.3)
Ease of use	3.8 (0.4)	4.4 (0.4)	4.1 (0.4)
Usefulness	4.4 (0.2)	4.5 (0.4)	4.1 (0.1)
Communication	4.4 (0.5)	4.4 (0.3)	4.2 (0.3)
Efficiency	3.2 (0.2)	3.7 (0.3)	3.7 (0.4)
Empowerment	3.8 (0.2)	4.4 (0.4)	3.5 (0.3)
Quality of care	4.0 (0.5)	4.1 (0.6)	3.9 (0.5)

^a^User satisfaction of usability attributes was rated on a scale of 1 (low agreement) to 5 (high agreement).

### User Statistics of End-of-Study Survey Respondents

**Figure 1:**
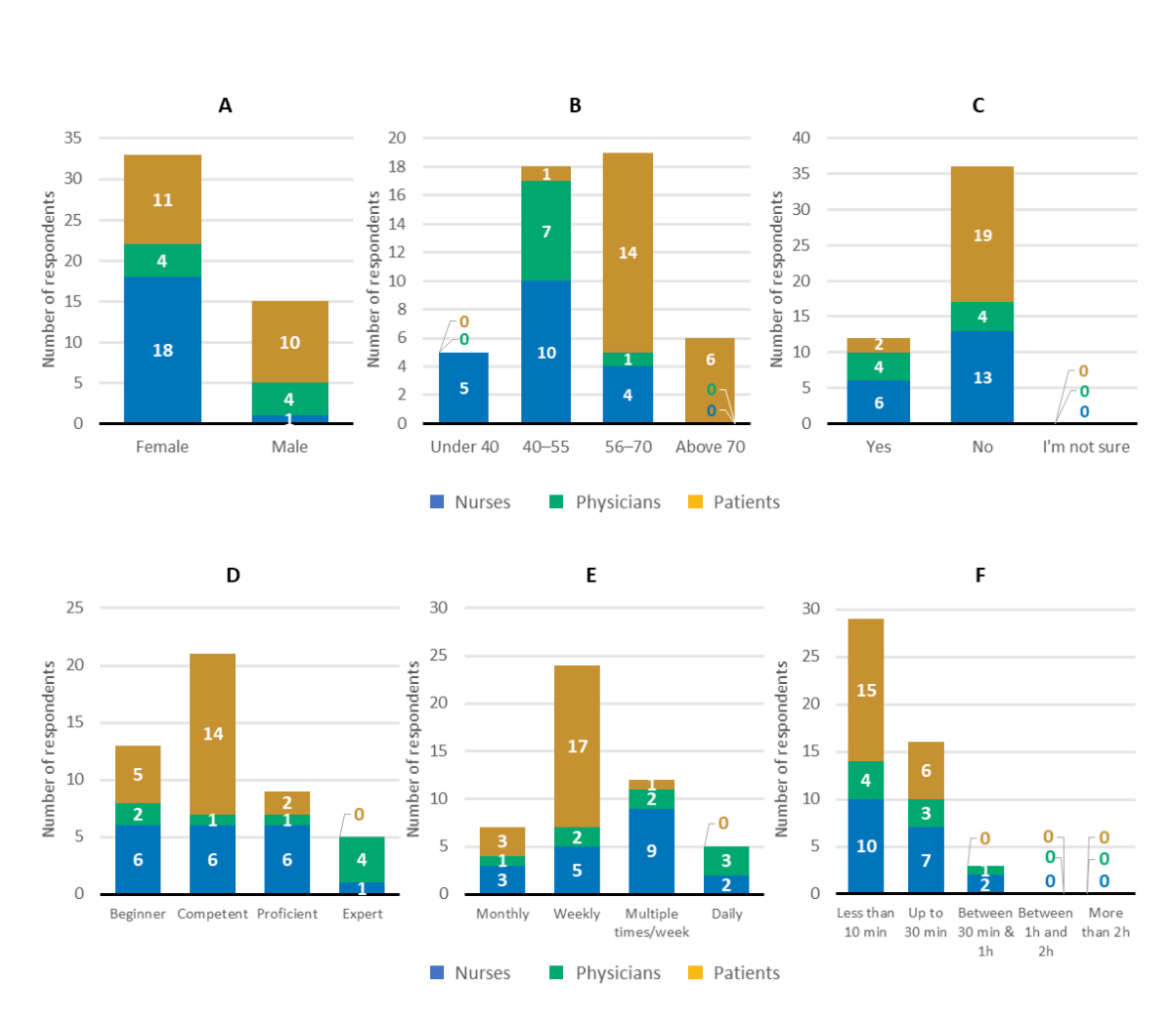
Characteristics of end-of-study survey respondents and digital patient monitoring and management (DPMM) tool usage (N=48: 19 [40%] nurses, 8 [17%] physicians, and 21 [44%] patients). (A) gender, (B) age, (C) DPMM use (ie, response to "Have you used Kaiku Health or other similar digital monitoring tools before this pilot?"), (D) level of proficiency (ie, response to "How would you rate your current proficiency level with regard to Kaiku Health?"), (E) usage rate (ie, response to "How often do you use Kaiku Health?"), and (F) time spent per session using the tool for clinicians and patients. Data are the number of respondents who provided the given response.

There were 48 respondents of the end-of-study survey: 19 (40%) nurses, 8 (17%) physicians, and 21 (44%) patients. Characteristics and information on tool usage are provided in Figure 1. Most respondents were female, primarily due to the higher number of participating female nurses (see Figure 1, A). Most respondents were 40 to 70 years old and had no previous experience of using Kaiku Health or other DPMM tools before their involvement in this pilot study (see Figure 1, B and C). Overall, 35 out of 48 (73%) end-of-study survey respondents considered their proficiency level during tool use to be competent, proficient, or expert (see Figure 1, D). Frequency of tool use was at least weekly for 41 out of 48 (85%) respondents (see Figure 1, E), with 29 out of 48 (60%) respondents indicating that they used the tool for 10 minutes or less per session (see Figure 1, F).

### Effect of the Tool on Communication, Quality of Patient Care, and Efficiency

End-of-study survey respondents were questioned about their perceptions of the effect of the tool on communication, quality of patient care, and efficiency. All user groups agreed that the tool facilitated more efficient and focused discussions between patients and HCPs: mean ratings ranged from 4.2 for nurses (SD 0.8) and patients (SD 1.0) to 4.4 for physicians (SD 0.8) (see Table 2).

**Table 2:** Effect of the digital patient monitoring and management tool on patient–health care professional communication for end-of-study survey respondents.

Survey statement	Rating^a^, mean (SD)		
	Nurses (n=19)	Physicians (n=8)	Patients (n=21)
Kaiku Health allows for more efficient communication with patients	4.2 (0.8)	4.4 (0.8)	N/A^b^
Kaiku Health helps to focus my discussions with my care team	N/A	N/A	4.3 (1.0)
Kaiku Health makes it easier to communicate with my care team	N/A	N/A	4.2 (1.0)

^a^Responses to survey statements were given on a scale of 1 (low agreement) to 5 (high agreement).

^b^N/A: not applicable. This user group was not presented with this statement.

Patients also indicated that the tool made communication with their care team easier: mean rating 4.3 (SD 1.0) (see Table 2). In that regard, the tool included a chat function that allowed for messages to be sent between patients and HCPs; overall, German or Finnish HCPs sent more messages to patients (n=326) than patients sent to German or Finnish HCPs (n=265), whereas the numbers of messages sent between Swiss HCPs and patients were similar: 47 versus 54 (see Multimedia Appendix 5).

Ratings from HCPs at the end-of-study survey showed that they believed that the tool helped to improve quality of patient care (mean rating 4.10, SD 0.85), permitting tailored discussions with patients (mean rating 4.35, SD 0.65), and that the symptom alert feature allowed earlier detection of symptoms (mean rating 4.25, SD 0.85) and tailoring of treatment plans (mean rating 3.9, SD 1.0). The self-care instructions function was appreciated by both HCPs and patients (mean ratings ranged from 4.0, SD 1.0, for physicians to 4.1, SD 0.9, for nurses and 4.1, SD 0.5, for patients). Patients also agreed that the tool made them feel more taken care of (mean rating 3.9, SD 1.3) (see Table 3).

**Table 3:** Effect of the digital patient monitoring and management tool on quality of patient care for end-of-study survey respondents.

Survey statement	Rating^a^, mean (SD)		
	Nurses (n=19)	Physicians (n=8)	Patients (n=21)
Kaiku Health helps me to improve quality of patient care	3.9 (1.0)	4.3 (0.7)	N/A^b^
Kaiku Health helps me tailor my discussions with my patients	4.2 (0.8)	4.5 (0.5)	N/A
The symptom alert feature alerts my staff to react to symptoms earlier	3.9 (1.2)	4.6 (0.5)	N/A
The symptom alert feature enables my staff to tailor treatment plans	3.8 (1.0)	4.0 (1.0)	N/A
Self-care instructions are valuable	4.1 (0.9)	4.0 (1.0)	N/A
Self-care instructions make me feel informed	N/A	N/A	4.1 (0.5)
Kaiku Health makes me feel more taken care of	N/A	N/A	3.9 (1.3)

^a^Responses to survey statements were given on a scale of 1 (low agreement) to 5 (high agreement).

^b^N/A: not applicable. This user group was not presented with this statement.

HCPs, particularly physicians, thought that the standalone tool was well integrated into their daily clinical workflow (mean rating 3.80, SD 0.75). They thought that it could help to improve efficiency by enabling workflow optimization between physicians and nurses (mean rating 3.75, SD 0.80) and freeing up time by decreasing the need for phone consultations (mean rating 3.75, SD 1.00) and patient visits (mean rating 3.45, SD 1.20) through online symptom assessment (see Table 4). Patients also thought that the tool improved efficiency by improving their ability to evaluate whether their symptoms required an unscheduled outpatient appointment (mean rating 3.9, SD 1.2) through prompts to contact HCPs regarding severe symptoms. Patients further reported a shortening of the time between health consultation requests and responses (mean rating 3.7, SD 1.2) (see Table 4).

**Table 4:** Effect of the digital patient monitoring and management tool on efficiency for end-of-study survey respondents: general comments.

Survey statement	Rating^a^, mean (SD)		
	Nurses (n=19)	Physicians (n=8)	Patients (n=21)
Kaiku Health is well integrated into my daily workflow	3.6 (1.0)	4.0 (0.5)	N/A^b^
The workflow management function between nurses and oncologists enables workflow optimization	3.4 (0.9)	4.1 (0.7)	N/A
Kaiku Health potentially decreases unnecessary patient visits and frees up time	3.4 (1.0)	3.5 (1.4)	N/A
Kaiku Health potentially decreases unnecessary patient phone calls and frees up time	3.4 (1.0)	4.1 (1.0)	N/A
Kaiku Health shortens the time between my health consultation requests and response	N/A	N/A	3.7 (1.2)
Kaiku Health helps me to better evaluate if my symptoms require a hospital visit	N/A	N/A	3.9 (1.2)

^a^Responses to survey statements were given on a scale of 1 (low agreement) to 5 (high agreement).

^b^N/A: not applicable. This user group was not presented with this statement.

For HCPs, the tool required little time for patient introduction, with most (18/27, 67%) taking up to 30 minutes for onboarding per patient. The tool also saved them time during patient visits (6/27, 22%, saved ≤5 minutes per consultation; 5/27, 19%, saved 6-10 minutes; and 1/27, 4%, saved 11-15 minutes; see Table 5) . Out of 21 patients, 3 (14%) reported that their need for an unscheduled, symptom-related hospital visit decreased per month during their use of the tool, while 1 patient (5%) reported an increased number of monthly visits and 8 (38%) reported no change in the frequency of unscheduled hospital visits. Out of 21 patients, 7 (33%) reported a decreased need for a phone consultation while using the tool; for 9 patients (43%), the need stayed the same (see Table 5). 

**Table 5:** Effect of the digital patient monitoring and management tool on efficiency for end-of-study survey respondents: health care professional time invested or saved, and patient need for unscheduled hospital visits and telephone consultations.

Survey question or statement and responses		Number of respondents who provided the given response, n (%)^a^		
		Nurses (n=19)	Physicians (n=8)	Patients (n=21)
**How long does it take to onboard the patient?**				
	Up to 30 minutes	13 (68)	5 (63)	N/A^b^
	Between 30 minutes and 1 hour	2 (11)	0 (0)	N/A
	Between 1 and 2 hours	0 (0)	0 (0)	N/A
	More than 2 hours	0 (0)	0 (0)	N/A
	I don’t onboard patients	4 (21)	3 (38)	N/A
**Kaiku Health allows me to save time, which amounts to approximately...**				
	Up to 5 minutes per consultation	3 (16)	3 (38)	N/A
	Between 6 and 10 minutes per consultation	4 (21)	1 (13)	N/A
	Between 11 and 15 minutes per consultation	0 (0)	1 (13)	N/A
	More than 16 minutes per consultation	0 (0)	0 (0)	N/A
	It does not save any time	5 (26)	2 (25)	N/A
	Kaiku Health needs even more time	1 (5)	0 (0)	N/A
	I am not sure	6 (32)	1 (13)	N/A
**Since I started using Kaiku Health, the number of unscheduled hospital visits due to observed symptoms...**				
	Decreased on average by 1 visit per month	N/A	N/A	1 (5)
	Decreased on average by 2 visits per month	N/A	N/A	2 (10)
	Decreased on average by 3 or more visits	N/A	N/A	0 (0)
	Increased on average by 1 visit per month	N/A	N/A	1 (5)
	Increased on average by 2 visits per month	N/A	N/A	0 (0)
	Increase on average by 3 or more visits	N/A	N/A	0 (0)
	Amount of visits did not change	N/A	N/A	8 (38)
	I don’t know or not applicable since I started	N/A	N/A	9 (43)
**Due to the use of Kaiku Health, my need to request a telephone consultation...**				
	Increased	N/A	N/A	0 (0)
	Decreased	N/A	N/A	7 (33)
	Stayed the same	N/A	N/A	9 (43)
	Does not apply	N/A	N/A	5 (24)

^a^Percentages may not add up to 100% due to rounding.

^b^N/A: not applicable. This user group was not presented with this question or statement.

### Exploration of HCP Needs, Expectations, and Perceived Value of the DPMM Tool

To gain qualitative insights into the needs, expectations, and experiences of HCPs with the tool, 19 HCPs—11 (58%) nurses and 8 (42%) physicians—were interviewed with open-ended questions at study end. Generally, expectations highlighted by HCPs were met or exceeded; improved efficiency and quality of patient care were the most prominent expectations of the tool and were mentioned by 8 out of 19 (42%) and 7 out of 19 (37%) interviewees, respectively (see Table 6 as well as Multimedia Appendix 6 for sample participant quotes). Improved efficiency and quality of patient care were also deemed the most value-adding attributes and were mentioned by 10 out of 19 (53%) interviewees for each attribute. Quotes highlighting improvements in efficiency and quality of patient care while using the tool are provided in Textboxes 1 and 2, respectively. Workload was the most prominent challenge of using the tool, as mentioned by 12 out of 19 (53%) interviewees, with some participants stating that the extra time needed to manage the standalone tool and enter the data sometimes compromised the efficiencies and time savings achieved elsewhere (see Table 6). Interoperability and system integration issues, as mentioned by 3 out of 19 (16%) interviewees but unlinked to tool functionality, were tightly related to workload challenges and could be considered the main cause for the perceived extra time (see Table 6).

**Table 6:** Expectations and perceived added value of the digital patient monitoring and management (DPMM) tool from qualitative health care professional interviews.

Expectations and perceptions of the DPMM tool		Expectation, perception, value, or challenge	Interviewees who mentioned the given theme (n=19)^a^, n (%)
**Expectations before the study**			
	Efficiency	Positive	8 (42)
	Quality of care	Positive	7 (37)
	Data generation	Positive	2 (11)
	This is the future	Positive	2 (11)
	Better patient education	Positive	1 (5)
	More transparency about patients’ symptoms	Positive	1 (5)
	Skepticism at the beginning	Neutral or negative	3 (16)
	Expected more from the drug- and indication-specific cancer immunotherapy module	Neutral or negative	1 (5)
**Perceived added value at the end of the study**			
	Efficiency	Key value attribute	10 (53)
	Quality of care	Key value attribute	10 (53)
	Communications and collaboration	Key value attribute	8 (42)
	Workflow	Key value attribute	8 (42)
	Empowerment	Key value attribute	5 (26)
	Workload	Challenge	12 (63)
	Interoperability and integration	Challenge	3 (16)

^a^Out of 19 interviewees, 11 (58%) were nurses and 8 (42%) were physicians.

**Quotes from two health care professionals (HCPs) to emphasize improvements in efficiency from using the digital patient monitoring and management tool.**

- “When the patient came up with a problem, we were all prepared for it and the patient was there for a shorter amount of time because we already knew how to respond in advance. That was an improvement.” [HCP interviewee #13]
- “You could deal with problems beforehand. I had already seen what has been discussed, what she herself has said, and then you could just go from there. That then ultimately leads to shorter, more concise consultation times.” [HCP interviewee #19]

**Quotes from three health care professionals (HCPs) to emphasize improvements in quality of patient care from using the digital patient monitoring and management tool.**

- “It became evident that we can use it for a more structured follow-up of the side effects of the treatments and symptoms of the patients.” [HCP interviewee #15]
- “For the data quality, for the care, it is a benefit. Undoubtedly. And they all felt really well cared-for, no doubt about it.” [HCP interviewee #13]
- “Perhaps some points are red [high severity] or newly yellow [medium severity], then you can say, ‘They must come to therapy earlier so that the doctor can talk to them directly and decide whether they would need only/either treatment or perhaps even hospitalization.’” [HCP interviewee #14]

### Use of the DPMM Tool’s Individual Functions

Among HCPs who responded to the end-of-study survey, the most commonly appreciated functions of the tool were the patient symptom alerts (26/27, 96%) and the direct message communication function between patients and HCPs (19/27, 70%; see Table 7). Other important features for HCPs were the ability to use the tool during patient consultations (15/27, 56%), the facilitation of more effective conversations and referrals between nurses and physicians through the triage function (13/27, 48%), the ability to use the tool during telephone consultations (13/27, 48%), and the onboarding of patients (13/27, 48%) (see Table 7).

Results from the end-of-study survey showed that patients had a similar preference with regard to the tool’s functions; the most commonly appreciated functions were the symptoms questionnaire (20/21, 95%) and the direct message communication function between HCPs and patients (9/21, 43%). The patient card content—a PDF that could be completed digitally—offered to patients treated with atezolizumab was also appreciated; 2 of the 4 (50%) patients responding to the survey used the function (see Table 7). The median time to fill out the symptom questionnaire ranged from 2 minutes and 18 seconds (Clinic D, Germany) to 9 minutes and 56 seconds (Clinic J, Germany); the mean median time for questionnaire completion was 4 minutes and 14 seconds (see Multimedia Appendix 7).

**Table 7:** Functions of the digital patient monitoring and management (DPMM) tool most commonly appreciated by end-of-study survey respondents.

Functions of the DPMM tool	Nurses (n=19), n (%)	Physicians (n=8), n (%)	Patients (n=21), n (%)
Monitor the patient’s symptoms	18 (95)	8 (100)	N/A^a^
Analyze the collected patient information	2 (11)	4 (50)	N/A
Draw patient reports	2 (11)	1 (13)	N/A
Directly communicate with the patients	14 (74)	5 (63)	N/A
Have more effective conversations and referrals between nurses and physicians	8 (42)	5 (63)	N/A
Use the tool during patient consultations	9 (47)	6 (75)	N/A
Use the tool during telephone consultations	7 (37)	6 (75)	N/A
Onboard patients to the tool	10 (53)	3 (38)	N/A
Symptoms questionnaire	N/A	N/A	20 (95)
Chat function	N/A	N/A	9 (43)
Patient card (n=4)^b^	N/A	N/A	2 (50)
Educational material^c^	N/A	N/A	3 (14)

^a^N/A: not applicable. This function was not relevant to this user group.

^b^Patient cards were part of the atezolizumab-specific material and were only relevant to patients treated with atezolizumab, of whom 4 responded to the survey.

^c^Educational material was offered to all 21 patients, although atezolizumab-specific educational material was offered only to atezolizumab-treated patients.

### Impact of Individual Functions of the DPMM Tool on Users

Ratings from the end-of-study survey respondents demonstrated that the tool empowered patients, helping them to feel more in control (patient mean rating 3.9, SD 1.2), increasing their feelings of safety during their treatment (patient mean rating 3.9, SD 1.2), and helping them to feel more secure in evaluating their symptoms (patient mean rating 3.8, SD 1.3) (see Multimedia Appendix 8). HCPs appreciated the compact overview of patient development offered by the dashboard (HCP mean rating 4.25, SD 0.70) (see Multimedia Appendix 8).

Overall, according to in-module activities, the drug- and indication-specific educational material within the tool was engaged by, based on the number of downloads, 80% of patients (36/45) (see Multimedia Appendix 9), with two of the three atezolizumab-specific material items engaged by all 4 of the patients treated with atezolizumab who responded to the survey. Total median article viewing time across all clinics for the drug- and indication-specific educational material was approximately 3.5 hours; the longest viewing time was observed for the breathing exercises video, which was 1 hour, 28 minutes, and 56 seconds (see Multimedia Appendix 10). According to the end-of-study survey, the educational material was found by all users to be very helpful and informative, especially the lung cancer material (mean user rating 4.30, SD 0.73), the breathing exercises video (mean user rating 4.20, SD 0.93), and the CIT video (mean user rating 4.1, SD 0.7) (see Multimedia Appendix 8). The atezolizumab-specific material (ie, patient card, information on preparing for first infusion and treatment, and medication-specific material) received the highest rating of any of the materials offered; all 4 atezolizumab-treated patients who responded to the survey rated it as 5 (see Multimedia Appendix 8).

### Data Sharing Statement

Qualified researchers may request access to analysis data via the corresponding author.

## Discussion

### Principal Findings

The CIT+ module that we co-developed with HCPs and patients was tested by physicians, nurses, and patients who considered themselves competent, proficient, or expert users. Most of them used the tool weekly (patients) or multiple times per week (HCPs), and most used the tool for 10 minutes or less per session. As this was a pilot study, a population sample representative of the general cancer population was not its purpose.

Overall, the results of our proof-of-concept pilot study demonstrate that user acceptance of the tool was high, with usefulness and communication being the most appreciated attributes. Mean ratings were consistently over 3.5, which, similar to previous studies, were assumed to indicate high agreement [60]. A pilot study assessing the Diabetes Family Teamwork Online intervention in patients with type 1 diabetes also used a Likert scale, but a 7-point scale rather than a 5-point scale [61]. However, similarly, the study deemed approximately 70% of the maximum score to equal high feasibility [61]. In this study, the symptom questionnaire and symptom alerts were the most commonly used functions of the DPMM tool among patients and HCPs, respectively, followed by the direct message communication function and the drug- and indication-specific educational material. The symptom alert function was a key element, enabling HCPs to define alerts for particular symptoms and severity. HCPs stated that this enabled them to detect and manage critical symptoms earlier and personalize treatment plans, a result aligned with findings from other similar studies [24,62,63]. Our results highlight the essential features that a DPMM tool should include to better serve the needs of both patients and HCPs in clinical practice. Currently, few available tools combine all these features [20].

All survey respondents agreed that the attributes of the DPMM tool enabled more efficient and focused communication between patients and HCPs; this positive impact on communication has also been reported in previous studies [38,51,62]. Furthermore, the tool empowered patients, which has been shown to be correlated with an improved QoL [64,65], and helped them to evaluate and monitor their symptom progression.

In general, HCPs believed that the standalone tool was well integrated in their daily clinical workflow and improved efficiency within the health care team. This is based on positive insights from interviews (see Table 6), where HCPs reported improvements in quality of care and communications with the care team as well as time savings in patient visits with tool use, allowing for more clinically meaningful time with patients. Such positive insights are consistent with previous similar studies [66-68]. However, in this study, time savings were sometimes compromised by interoperability issues, such as lack of tool integration into the information technology (IT) system of the clinic and, consequently, data having to be gathered from both the web-based Kaiku Health platform and the clinic IT system. This challenge has been demonstrated in numerous other studies [38,40,69-71]. However, it should be resolved once the interoperability and system integration are incorporated beyond the pilot study as participating clinics and hospitals undertake complete rollout.

Notably, in addition to the time savings reported per patient visit by some HCPs, the tool also has the potential to free up time by decreasing the need for unscheduled outpatient appointments and telephone consultations, as reported by some patients (see Table 5). This is consistent with a possible reduction of scheduled visits in lung cancer patients following use of a DPMM tool that enables early detection of critical symptoms [23]. Considering most HCPs invested up to 30 minutes in introducing patients to the tool, and half of them saved 5 minutes or more per consultation, the time invested in patient onboarding was repaid within a few visits. The time saved through use of the DPMM tool can be invested in addressing other patient needs or serving more patients, highlighting the health-economic benefits of the tool.

Compared with studies of other ePRO-based tools, the CIT+ tool studied here showed similar high feasibility, patient engagement, and patient satisfaction [62,72-74]. However, the CIT+ module also harnesses features not commonly seen in other tools. For example, a 2019 systematic review of existing electronic symptom reporting systems developed for patients during cancer treatment found that fewer than half included a feature for delivering advice to patients in symptom self-management, and fewer than a third gave patients access to general educational information [20]. An even less common feature was the facility to support patient-HCP communication (15%) [20]. The CIT+ tool currently harnesses all these features, which is pertinent considering that previous research has indicated communication features, in particular, to be highly valued and utilized by patients [75-77].

Based on the results of this pilot study and insights from other studies, we propose several recommendations for future use in clinical practice or in study settings. For both, a positive and easy user experience is essential (eg, via optimization of the user interface), thereby enabling a choice of symptoms to be reported, as is providing automated contextual information according to user needs. In a clinical practice setting, a higher workflow efficiency and a much earlier detection of critical symptoms could be achieved with a seamless integration into the clinical workflow. Hence, integration into the local electronic health record system would be beneficial but may require substantial initial investments, given the complexities of existing systems. Such an integration may also enable local integration of patient self-management information and materials; these materials have been shown to be critical in supporting patient self-monitoring [77-79]. Integration may further enable connections to local resources and services, such as mental health or other supportive services, which can improve personalization of treatment according to patient needs and local standards. An enhanced local integration and tailoring to patient needs may improve adherence and allow patients to review and monitor their own data, which is the case for barely half of current ePRO systems [20].

In a clinical study setting, where assessment standards can be defined centrally and where stratified randomization of patients into standard-of-care control cohorts is possible, it is of interest to assess impact of the DPMM tool on patient outcomes and health care resource utilization and subsequently optimize for these elements. Regarding patient outcomes, assessment of QoL [22,23], in particular time to deterioration of symptoms and symptom development or adverse event intensity and duration over time, are important. Patients’ abilities to work, their mental well-being, their self-care needs, and their self-efficacy are also important to assess. In regard to health care resource utilization, assessment of hospitalization days and emergency room visits [22], duration and adherence of treatment, co-medication use, and supportive care costs could yield valuable insights into the impact of a DPMM tool on clinical practice.

### Limitations of the Study

A key limitation of this pilot study was its small sample size and single-arm design. Also, due to disease severity, the study was only able to recruit one participant for the patient advisory board. Further to this, DPMM tools are currently rarely used in routine clinical practice; as a result, there could be a bias for inclusion of sites, HCPs, and patients who are more accepting of these tools from the outset and are, therefore, more positive about their use. There are also several limitations associated with the chosen methods of data collection in this pilot study, namely surveys and interviews. Both rely on memory for answering questions, which may affect accuracy of the information received; furthermore, some bias may be created as participants who chose not to respond to survey questions or to participate in HCP interviews may have had different opinions from those who did so. Closed-ended questions, like those in our surveys, may have lower validity than open-ended questions, and answer options may be subject to the interpretation of different respondents. Finally, as discussed above, our DPMM tool was not integrated into the local electronic health record and patient management IT systems but was used as a standalone tool, which led to patient information needing to be gathered and recorded twice, causing additional workload for HCPs. This may have impeded HCPs from having an even more positive experience with the DPMM tool, thereby hampering their positive perceptions of the impact of the tool on clinical workflow efficiency. Hence, integration of the DPMM tool into the clinical IT data flow will be an important aspect of efficient routine clinical practice in the future. These limitations will be addressed in planned studies to evaluate the impact of the DPMM tool on patient health and health-economic benefits in a broader, better implemented, and more comprehensive approach.

### Conclusions

Our results demonstrate high user satisfaction and acceptance of DPMM tools by HCPs and patients and highlight the contributions that DPMM tools can make to clinical care of patients with advanced or metastatic NSCLC treated with CIT monotherapy. Findings here will offer an incentive for continuous improvement and development of our tool, so that a platform can be provided that best serves the needs of HCPs and patients in this and other indications. The results add to the growing evidence base that DPMM tools can improve management of patients with cancer, empower patients, and have a health-economic impact by saving time in visits and reducing the need for patient telephone consultations [21,22]. Improvements in patient care have also been observed following the introduction of DPMM tools in other disease areas, such as multiple sclerosis [80-82]. Further studies or registries that allow investigation of the use of our DPMM tool may provide insights into whether its use would have any significant effect on other outcome measures, such as patient survival or QoL, or on health-economic benefits.

## Appendix

Multimedia Appendix 1 Video clip showing how participants used the tool.

Multimedia Appendix 2 The composition and size of the trained care teams.

Multimedia Appendix 3 Method of thematic analysis.

Multimedia Appendix 4 Preferred platform for tool usage in each of the user groups. The percentage indicates the absolute percentage distribution of all the log-ins for each device for each user group.

Multimedia Appendix 5 The number of chat messages sent between patients and health care professionals (HCPs) per clinic.

Multimedia Appendix 6 Participant quotes highlighting some of the main expectations before the pilot study.

Multimedia Appendix 7 Table showing median time to fill out the symptom questionnaire per clinic.

Multimedia Appendix 8 End-of-study survey respondents’ experiences of different features of the digital patient monitoring and management (DPMM) tool (N=48: 19 [40%] nurses, 8 [17%] physicians, and 21 [44%] patients). Data are the means of the given values on a scale of 1 (minimum value) to 5 (maximum value) for that user group.

Multimedia Appendix 9 Patient engagement in disease- and medication-specific educational material.

Multimedia Appendix 10 Median reading times of drug- and indication-specific educational material.

## Abbreviations

ALK: anaplastic lymphoma kinase
BRAF: B-Raf proto-oncogene
CIT: cancer immunotherapy
CIT+: drug- and indication-specific cancer immunotherapy
CTCAE: Common Terminology Criteria for Adverse Events
DPMM: digital patient monitoring and management
EGFR: epidermal growth factor receptor
ePRO: electronic patient-reported outcome
HCP: health care professional
IT: information technology
MET: N-methyl-N'-nitroso-guanidine human osteosarcoma transforming
NCI: National Cancer Institute
NSCLC: non-small cell lung cancer
NTRK: neurotrophic tropomyosin receptor kinase
OS: overall survival
PD-L1: programmed death-ligand 1
PRO: patient-reported outcome
QoL: quality of life
RET: rearranged during transfection
ROS1: ROS proto-oncogene 1
TAM: technology acceptance model
