# Supplementary material for: Digital Monitoring and Management of Patients With Advanced or Metastatic Non-Small Cell Lung Cancer Treated With Cancer Immunotherapy and Its Impact on Quality of Clinical Care: Interview and Survey Study Among Health Care Professionals and Patients
Source: J Med Internet Res. 2020 Dec 21;22(12):e18655. doi: 10.2196/18655 (PMC7781800; doi:10.2196/18655)
Supplement: Multimedia Appendix 2 [file jmir_v22i12e18655_app2.docx]

## Multimedia Appendix 2

Table showing the composition and size of the trained care teams.

|  |  | Number of users trained  n | | |
| --- | --- | --- | --- | --- |
|  |  | Nurses | Physicians | Total |
| **Clinic, country** | |  |  |  |
|  | Clinic G, Switzerland | 6 | 3 | 9 |
|  | Clinic B, Finland | 3 | 2 | 5 |
|  | Clinic C, Finland | 3 | 2 | 5 |
|  | Clinic I, Germany | 3 | 2 | 5 |
|  | Clinic A, Germany | 2 | 2 | 4 |
|  | Clinic D, Germany | 2 | 2 | 4 |
|  | Clinic J, Germany | 1 | 2 | 3 |
|  | Clinic E, Switzerland | 1 | 1 | 2 |
|  | Clinic F, Finland | 0 | 2 | 2 |
|  | Clinic H, Finland | 1 | 1 | 2 |
| Total, n | | 22 | 19 | 41 |
