# Supplementary material for: Digital Monitoring and Management of Patients With Advanced or Metastatic Non-Small Cell Lung Cancer Treated With Cancer Immunotherapy and Its Impact on Quality of Clinical Care: Interview and Survey Study Among Health Care Professionals and Patients
Source: J Med Internet Res. 2020 Dec 21;22(12):e18655. doi: 10.2196/18655 (PMC7781800; doi:10.2196/18655)
Supplement: Multimedia Appendix 3 [file jmir_v22i12e18655_app3.docx]

## Multimedia Appendix 3

**Method of thematic analysis**

The study followed the steps of thematic analysis as described by Braun and Clarke [1, 2]. After the initial analysis and coding by Christine Jacob, this was reviewed by Manuel Kammermann, and any cases of disagreement were discussed in conjunction with Alexander Klein and mutually agreed upon. The graphs are used to emphasize frequency by showing visually which themes were brought up by more participants than others. The frequency reflected in the visuals counts the theme only once per participant and doesn’t accumulate if the same participant brought the same theme up several times. Such visualization mainly aims to improve the comprehension of the article especially when contrasting two elements [3], and can provide a clear and simple illustration of the dominant themes and ideas for lay readers [4].

**References**

1. Braun V, Clarke V (2006) Using thematic analysis in psychology Qual Res Psychol 3:77-101. 10.1191/1478088706qp063oa

2. Braun V, Clarke V (2014) What can "thematic analysis" offer health and wellbeing researchers? Int J Qual Stud Health Well-being 9:26152. 10.3402/qhw.v9.26152

3. Verdinelli S, Scagnoli NI (2013) Data Display in Qualitative Research International Journal of Qualitative Methods 12:359-381. 10.1177/160940691301200117

4. Henderson S, Segal EH (2013) Visualizing Qualitative Data in Evaluation Research New Directions for Evaluation 2013:53-71. 10.1002/ev.20067
