# Supplementary figures and images for: Digital Monitoring and Management of Patients With Advanced or Metastatic Non-Small Cell Lung Cancer Treated With Cancer Immunotherapy and Its Impact on Quality of Clinical Care: Interview and Survey Study Among Health Care Professionals and Patients
Source: J Med Internet Res. 2020 Dec 21;22(12):e18655. doi: 10.2196/18655 (PMC7781800; doi:10.2196/18655)

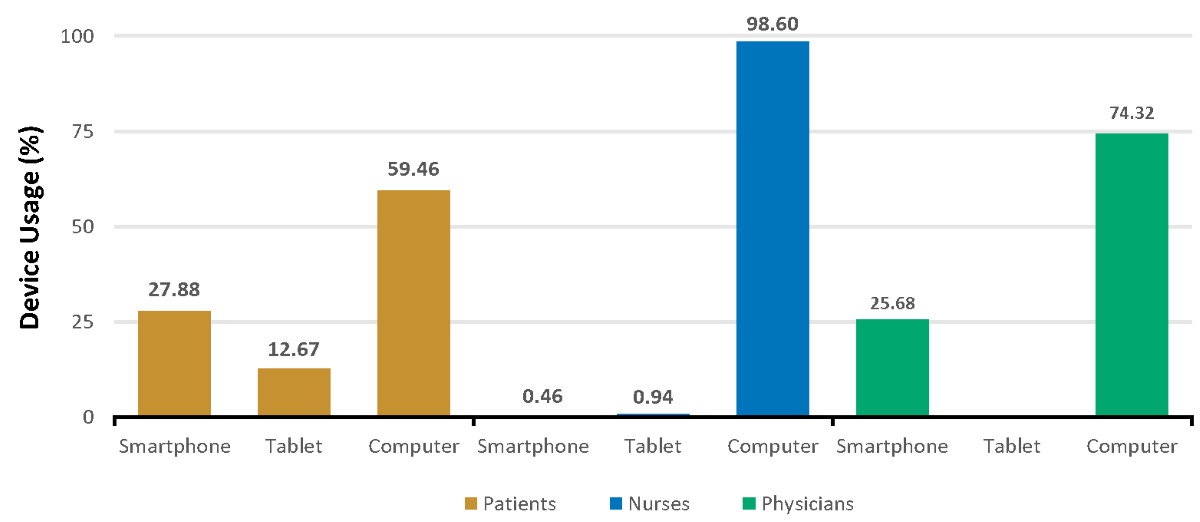

Supplement: Multimedia Appendix 4 [file jmir_v22i12e18655_app4.png]

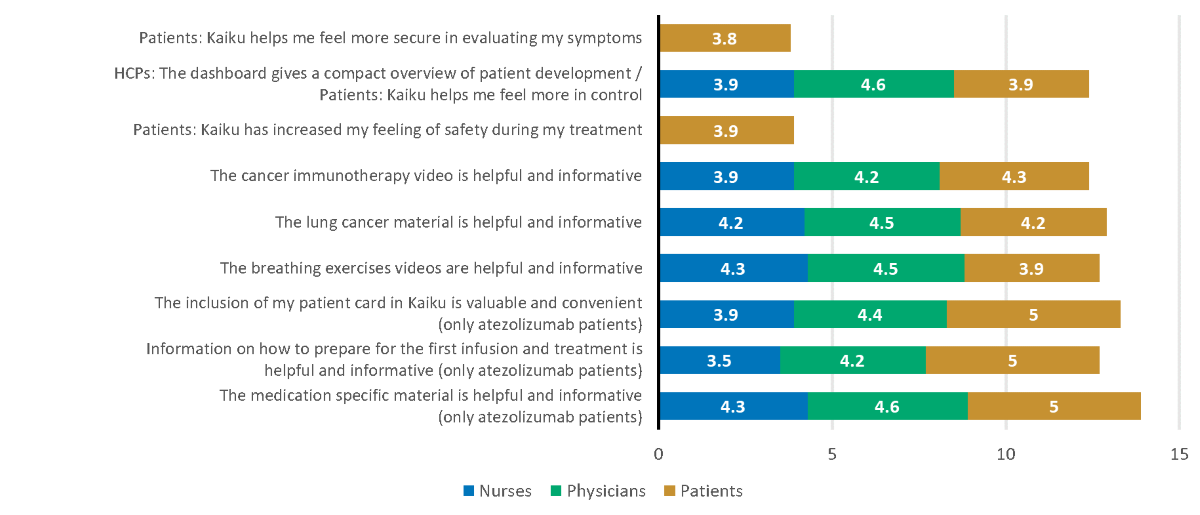

Supplement: Multimedia Appendix 8 [file jmir_v22i12e18655_app8.png]
