# Supplementary material for: Digital Monitoring and Management of Patients With Advanced or Metastatic Non-Small Cell Lung Cancer Treated With Cancer Immunotherapy and Its Impact on Quality of Clinical Care: Interview and Survey Study Among Health Care Professionals and Patients
Source: J Med Internet Res. 2020 Dec 21;22(12):e18655. doi: 10.2196/18655 (PMC7781800; doi:10.2196/18655)
Supplement: Multimedia Appendix 5 [file jmir_v22i12e18655_app5.docx]

## Multimedia Appendix 5

Table showing the number of chat messages sent between patients and HCPs per clinic.

|  |  | Active patients  n | Number of messages sent by patients | Number of messages sent by HCPs |
| --- | --- | --- | --- | --- |
|  |  |  |  |  |
| **Clinic, country** | |  |  |  |
|  | Clinic A, Germany | 10 | 70 | 72 |
|  | Clinic B, Finland | 4 | 58 | 63 |
|  | Clinic C, Finland | 3 | 49 | 74 |
|  | Clinic D, Germany | 4 | 48 | 73 |
|  | Clinic E, Switzerland | 10 | 40 | 32 |
|  | Clinic F, Finland | 2 | 29 | 29 |
|  | Clinic G, Switzerland | 4 | 14 | 15 |
|  | Clinic H, Finland | 4 | 6 | 9 |
|  | Clinic I, Germany | 3 | 5 | 5 |
|  | Clinic J, Germany | 1 | 0 | 1 |
| Total, n | | 45 | 319 | 373 |
