# Supplementary material for: Digital Monitoring and Management of Patients With Advanced or Metastatic Non-Small Cell Lung Cancer Treated With Cancer Immunotherapy and Its Impact on Quality of Clinical Care: Interview and Survey Study Among Health Care Professionals and Patients
Source: J Med Internet Res. 2020 Dec 21;22(12):e18655. doi: 10.2196/18655 (PMC7781800; doi:10.2196/18655)
Supplement: Multimedia Appendix 6 [file jmir_v22i12e18655_app6.docx]

## Multimedia Appendix 6

Table of participant quotes highlighting some of the main expectations before the pilot study.

| **Theme** | **Participant quotes** |
| --- | --- |
| Data generation | “That it would generate additional information that we normally do not get from patients.”  (HCP interviewee number 14) |
| This is the future | “We thought that this is very much up to date, and this is something where, in the very short future where maybe every patient is going to be a part of, or most of the patients are going to be followed through.”  (HCP interviewee number 18) |
| Better patient education | “I think it is a good way to give clinically, how to say, I’d say clinically validated data to patients” (HCP interviewee number 16) |
| More transparency about patient symptoms | “I guess just an overview of the transparency of side effects and immunotherapies.”  (HCP interviewee number 2) |
| Skepticism at the beginning | “The first thought was like that this was just one sort of an extra task, which didn’t yet, or I didn’t know if it would help or the opposite.”  (HCP interviewee number 9)  “I didn't really know a lot about it, I was simply prepared to go with the flow and actually I was completely and positively amazed.”  (HCP interviewee number 4) |
| Expected More from the CIT+ Module | “I would say that it does not differ that much from the Kaiku immunotherapy platform that we used earlier, so if I tell you the truth I kind of expected it to differ more.”  (HCP interviewee number 15) |
