# Supplementary material for: Digital Monitoring and Management of Patients With Advanced or Metastatic Non-Small Cell Lung Cancer Treated With Cancer Immunotherapy and Its Impact on Quality of Clinical Care: Interview and Survey Study Among Health Care Professionals and Patients
Source: J Med Internet Res. 2020 Dec 21;22(12):e18655. doi: 10.2196/18655 (PMC7781800; doi:10.2196/18655)
Supplement: Multimedia Appendix 7 [file jmir_v22i12e18655_app7.docx]

## Multimedia Appendix 7

Table showing median time to fill out the symptom questionnaire per clinic.

|  |  | Active patients  n | Median time to fill out symptom questionnaire  min:sec |
| --- | --- | --- | --- |
|  |  |  |  |
| **Clinic, country** | |  |  |
|  | Clinic F, Finland | 2 | 02:55 |
|  | Clinic G, Switzerland | 4 | 02:29 |
|  | Clinic D, Germany | 4 | 02:18 |
|  | Clinic B, Finland | 4 | 04:24 |
|  | Clinic C, Finland | 3 | 03:36 |
|  | Clinic E, Switzerland | 10 | 03:49 |
|  | Clinic A, Germany | 10 | 04:14 |
|  | Clinic H, Finland | 4 | 04:30 |
|  | Clinic J, Germany | 1 | 09:56 |
|  | Clinic I, Germany | 3 | 04:05 |
