# Supplementary material for: Digital Monitoring and Management of Patients With Advanced or Metastatic Non-Small Cell Lung Cancer Treated With Cancer Immunotherapy and Its Impact on Quality of Clinical Care: Interview and Survey Study Among Health Care Professionals and Patients
Source: J Med Internet Res. 2020 Dec 21;22(12):e18655. doi: 10.2196/18655 (PMC7781800; doi:10.2196/18655)
Supplement: Multimedia Appendix 9 [file jmir_v22i12e18655_app9.docx]

## Multimedia Appendix 9

Table of patient engagement in disease- and medication-specific educational material.

|  |  | Active Patients  n | Engagement with educational material  n (%) | | | | | | |
| --- | --- | --- | --- | --- | --- | --- | --- | --- | --- |
|  |  |  | Overall | Breathing exercise videos | Your CIT | What is NCSLC? | CIT+ ^a^  Infusion preparation | CIT+ ^a^  Patient card | CIT+ ^a^  Your treatment |
| **Clinic, country** | |  |  |  |  |  |  |  |  |
|  | Clinic E,  Switzerland, n (%) | 10 | 8/10  (80.0) | 5/10  (50.0) | 3/10  (30.0) | 4/10  (40.0) | 1/1  (100.0) | 1/1  (100.0) | 0 |
|  | Clinic A,  Germany,  n (%) | 10 | 8/10  (80.0) | 5/10  (50.0) | 5/10  (50.0) | 6/10  (60.0) | 2/2  (100.0) | 2/2  (100.0) | 2/2  (100.0) |
|  | Clinic C,  Finland,  n (%) | 3 | 5 ^b^ | 3/3  (100.0) | 3/3  (100.0) | 3/3  (100.0) | 0 | 0 | 0 |
|  | Clinic I,  Germany,  n (%) | 3 | 3/3  (100.0) | 1/3  (33.3) | 3/3  (100.0) | 1/3  (33.3) | 0 | 0 | 0 |
|  | Clinic D,  Germany,  n (%) | 4 | 3/4  (75.0) | 2/4  (50.0) | 2/4  (50.0) | 3/4  (75.0) | 1/1  (100.0) | 1/1  (100.0) | 1/1  (100.0) |
|  | Clinic B,  Finland,  n (%) | 4 | 3/4  (75.0) | 3/4  (75.0) | 3/4  (75.0) | 2/4  (50.0) | 0 | 0 | 0 |
|  | Clinic H,  Finland,  n (%) | 4 | 2/4  (50.0) | 1/4  (25.0) | 2/4  (50.0) | 1/4  (25.0) | 0 | 0 | 0 |
|  | Clinic F,  Finland,  n (%) | 2 | 2/2  (100.0) | 1/2  (50.0) | 1/2  (50.0) | 1/2  (50.0) | 0 | 0 | 0 |
|  | Clinic G,  Switzerland, n (%) | 4 | 1/4  (25.0) | 1/4  (25.0) | 1/4  (25.0) | 1/4  (25.0) | 0 | 0 | 0 |
|  | Clinic J,  Germany,  n (%) | 1 | 1/1  (100.0) | 1/1  (100.0) | 1/1  (100.0) | 0 | 0 | 0 | 0 |
| Total, n (%) | | 45 | 36/45 (80.0) | 23/45  (51.1) | 24/45 (53.3) | 22/45 (48.9) | 4/4  (100.0) | 4/4  (100.0) | 3/4  (75.0) |

Data are number and proportion of patients that read an article within the tool.

^a^CIT+: drug (atezolizumab)- and indication-specific cancer immunotherapy module.

^b^A total of seven patients registered at Clinic C but only three remained active at the time of data cut-off.
