# Supplementary material for: Digital Monitoring and Management of Patients With Advanced or Metastatic Non-Small Cell Lung Cancer Treated With Cancer Immunotherapy and Its Impact on Quality of Clinical Care: Interview and Survey Study Among Health Care Professionals and Patients
Source: J Med Internet Res. 2020 Dec 21;22(12):e18655. doi: 10.2196/18655 (PMC7781800; doi:10.2196/18655)
Supplement: Multimedia Appendix 10 [file jmir_v22i12e18655_app10.docx]

## Multimedia Appendix 10

Table of median reading times of drug- and indication-specific educational material.

|  | |  | Total median article reading time ^a^  hr:min:sec | | | | | |
| --- | --- | --- | --- | --- | --- | --- | --- | --- |
|  | | Patients who read an article  n | Breathing exercise videos | Your CIT | What is NCSLC? | CIT+ ^b^  Infusion preparation | CIT+ ^b^  Patient card | CIT+ ^b^  Your treatment |
| **Clinic, country** | |  |  |  |  |  |  |  |
|  | Clinic A, Germany | 8 | 00:07:05 | 00:20:15 | 00:07:42 | 00:01:58 | 00:00:14 | 00:01:52 |
|  | Clinic I, Germany | 3 | 00:11:47 | 00:09:18 | 00:34:27 | 00:00:00 | 00:00:00 | 00:00:00 |
|  | Clinic D, Germany | 3 | 00:44:48 | 00:08:58 | 00:02:42 | 00:00:00 | 00:00:11 | 00:00:00 |
|  | Clinic E, Switzerland | 8 | 00:10:30 | 00:07:48 | 00:01:36 | 00:00:23 | 00:00:25 | 00:00:00 |
|  | Clinic B, Finland | 3 | 00:02:00 | 00:05:09 | 00:01:20 | 00:00:00 | 00:00:00 | 00:00:00 |
|  | Clinic F,  Finland | 2 | 00:00:38 | 00:03:39 | 00:00:22 | 00:00:00 | 00:00:00 | 00:00:00 |
|  | Clinic C, Finland | 5 | 00:02:36 | 00:03:24 | 00:02:27 | 00:00:00 | 00:00:00 | 00:00:00 |
|  | Clinic H, Finland | 2 | 00:01:09 | 00:02:36 | 00:00:54 | 00:00:00 | 00:00:00 | 00:00:00 |
|  | Clinic J, Germany | 1 | 00:02:18 | 00:01:54 | 00:00:00 | 00:00:00 | 00:00:00 | 00:00:00 |
|  | Clinic G, Switzerland | 1 | 00:06:05 | 00:00:56 | 00:01:03 | 00:00:00 | 00:00:00 | 00:00:00 |
| Total | |  | 01:28:56 | 01:03:57 | 00:52:33 | 00:02:21 | 00:00:50 | 00:01:52 |

^a^Total article reading time was calculated by multiplying the number of patients that read the article with the median reading time.

^b^CIT+: drug (atezolizumab)- and indication-specific cancer immunotherapy module.
